# The Soybean GmNARK Affects ABA and Salt Responses in Transgenic *Arabidopsis thaliana*

**DOI:** 10.3389/fpls.2018.00514

**Published:** 2018-04-18

**Authors:** Chunhong Cheng, Changman Li, Diandong Wang, Lifeng Zhai, Zhaoming Cai

**Affiliations:** College of Life Science and Technology, Yangtze Normal University, Chongqing, China

**Keywords:** *GmNARK*, soybean, *Arabidopsis thaliana*, ABA, NaCl, abiotic stress

## Abstract

*GmNARK* (*Glycine max* nodule autoregulation receptor kinase) is the homolog of *Arabidopsis thaliana CLAVATA1* (*CLV1*) and one of the most important regulators in the process of AON (Autoregulation of Nodulation), a process that restricts excessive nodule numbers in soybean. However, except for the function in AON, little is known about this gene. Here, we report that GmNARK plays important roles in process of plant response to abiotic stresses. Bioinformatic analysis and subcellular localization experiment results showed that GmNARK was a putative receptor like kinase and located at membrane. The promoter of *GmNARK* contains manifold *cis* regulatory elements that are responsive to hormone and stresses. Gene transcript expression pattern analysis in soybean revealed *GmNARK* was induced by ABA and NaCl treatment in both shoot and root. Overexpression of GmNARK in *Arabidopsis* resulted in higher sensitivity to ABA and salt treatment during seed germination and greening stages. We also checked the expression levels of some ABA response genes in the transgenic lines; the results showed that the transcript level of all the ABA response genes were much higher than that of wild type under ABA treatment. Our results revealed a novel role of GmNARK in response to abiotic stresses during plant growth and development.

## Introduction

Plants are frequently exposed to various environmental stresses during their life time that greatly affect their growth and development (Liu et al., 2011). Abiotic stresses such as drought and high salinity significantly diminish plant growth, yield and distribution. To adapt to such detrimental conditions plants have evolved many innate strategies for survival, a key one being a metabolic process called ABA accumulation (Broun, 2004; Bohnert et al., 2006). Abscisic acid (ABA) is a key regulator of plant adaptation to stress and the ABA signaling pathway is composed of many important regulatory components and turnover mechanisms in response to constantly changing environmental conditions (Cheng et al., 2017). The hormone ABA accumulates during the stages of seedlings growth and plant maturation protecting plants from damage induced by drought, salinity levels, and pathogenic attack (Lopez-Molina et al., 2001; Finkelstein et al., 2002).

CLV1 is responsible for the progression of meristem cells toward organ initiation in *Arabidopsis*, an integral component of the receptor kinase signaling pathway alongside CLV2 and CLV3 (Clark et al., 1997; Schoof et al., 2000). Loss of function mutation of *CLV1* results in delayed organ initiation; this leads to accumulation of meristem cells and an increased size of the shoot meristem. The mutation of *clv1* is usually performed with enlarged inflorescence meristems and additional organs, which are mainly stamens and carpels (Clark et al., 1993).

CLV1 functions by repressing the expression of WUSCHEL (WUS), known for playing an important role in the maintenance of shoot and floral meristems. The homeostasis between CLV signaling and WUS is important for the normal operation of shoot organ differentiation (Schoof et al., 2000). The homolog of CLV1 in *Oryza sativa* is FON1 (FLORAL ORGAN NUMBER1); this gene encodes a leucine-rich repeat receptor kinase and plays a similar role as CLV1. Mutation of *FON1* shows increased numbers of reproductive organs, including lodicules, stamens and pistils. However, no detectable abnormalities are observed in the number of inflorescences and vegetative organs (Nagasawa et al., 1996; Suzaki et al., 2004). The homolog of CLV1 in maize is TD1 (Thick Tassel Dwarf 1); this gene possesses a similar protein structure to CLV1 and functions in the same way on the controlling of inflorescence meristems. Loss of function mutant *td1* shows thickening of the rachis, higher spikelet density, and extra glumes and stamens. Besides those, weak vegetative phenotype could be observed in *td1* mutant plants (Bommert et al., 2004). Taken alongside previous results, whether in monocots or dicots, the CLV signaling pathway for regulating meristem maintenance is conserved. On rare occasions other functions of CLV1 are uncovered in regulation of plant development.

GmNARK (*Glycine max* nodule autoregulation receptor kinase) is the homolog of *Arabidopsis* CLV1 in soybean, which was first cloned in the wild type and fasciation mutant of soybean (Yamamoto et al., 2000). However, the main function of GmNARK focuses on root development and AON (Autoregulation Of Nodulation) in soybean (Searle et al., 2003). Although GmNARK is the homolog of CLV1, no conserved function with respect to the regulation of inflorescence meristems was reported. Previous research suggested that *GmNARK* could be detected not only in root, but also in shoot tissue (Elise et al., 2005). As a leucine-rich repeat receptor-like kinase, GmNARK mainly functioned in the leaf. This protein accepted the CLE peptides derived from the root, following with the synthesis of SDI (shoot-derived inhibitor) in shoot, which was then transported to the root, leading to the inhibition of nodulation (Searle et al., 2003; Reid et al., 2011; Lim et al., 2011). In root, GmNARK also plays an important role in the inhibition of nodulation by root-derived and nitrate-induced CLE peptides (Reid et al., 2011, 2013; Lim et al., 2014; Mirzaei et al., 2017). Some permutations of the GmNARK loss of function (*nts1116* with a mutation of V837A and *nts1007* with a mutation of V370D) perform a function of AON, eventually leading to the hypernodulation and supernodulation phenotypes (Searle et al., 2003; Lin et al., 2010). Although GmNARK plays crucial roles in AON, rarely are proteins identified as interactive with this protein except GmKAPP. It has been reported that GmNARK could phosphorylate GmKAPP1 and GmKAPP2; subsequently GmKAPP1 and GmKAPP2 could dephosphorylate GmNARK (Miyahara et al., 2008). However, no impact on AON of GmKAPP were reported. To investigate functional downstream partners of GmNARK in nodulation signaling pathway, the transcriptome sequencing assay were performed using the leaves of wild-type and *nts1007* plants. The microarray result showed that GmNARK regulated the expression of genes in JA pathway indicating that GmNARK may participate in hormone signaling and plant defense (Kinkema and Gresshoff, 2008). No other functions of GmNARK were reported till now.

Our research found several stress responsive *cis*-elements in the promoter region of *GmNARK* by bioinformatic analysis; the expression of *GmNARK* was induced by salt and ABA in both root and shoot. Using subcellular localization analysis, we found that GmNARK-GFP was located in the cell membrane. Interestingly, the fusion protein was also detected in vesicles and trafficked via the endocytic pathway. Overexpression of *GmNARK* in *Arabidopsis* resulted in increased sensitivity to salt and ABA stresses during seed germination and cotyledon greening. These results suggest that GmNARK not only plays key roles in AON, but also is involved in response to abiotic stresses of plants. Our study will provide important and novel insights into the function of the soybean *GmNARK* and its potential roles in regulating plant response to ABA and salt.

## Materials and Methods

### Plant Materials and Growth Conditions

*Arabidopsis thaliana* ecotype (*Col-0*) and *Glycine max* [L.] Merrill cv. Williams 82 were used in this study. *Arabidopsis* seeds were surface sterilized and plated on MS medium (Sigma-Aldrich, St. Louis, MO, United States) with 1% sucrose and 8 g/L agar (Sigma-Aldrich, St. Louis, MO, United States). The seeds were chilled at 4°C in the dark for 2 days and then transferred to growth room at 22°C and 6000 lx under long-day conditions (16 h light/8 h dark). The seeds germination rate and seedlings greening rate were recorded at the specified time points. Soybean seeds Williams 82 were sowed in pots containing a 3:1 mixture of vermiculite and perlite and grown under 16 h/8 h light/dark at 25°C and 6000 lx with 50% relative humidity. After 4 days, the seedlings of Williams 82 were treated with BD liquid medium with or without 150 mM NaCl and 100 μM ABA, respectively. After 0, 3, 6, 12, and 24 h, the leaf and root materials were collected for RNA isolation, respectively.

### Phenotypic Analysis Assay

For phenotypic analysis under the treatment of ABA and NaCl, Seeds were sterilized and plated on MS medium containing 1 % sucrose with 150 mM NaCl or 0.25 μM ABA or not (three plates per treatment and 30 seedlings per line). The plates were transferred to chamber at 22°C and 6000 lx after stratification under long-day conditions (16 h light/8 h dark). The germination rate and greening rate were counted at the indicated time points.

### Bioinformatics Analysis

The protein sequences were alimented by ClustalX 1.83 (Thompson et al., 1997), and a phylogenetic tree was constructed using neighbor joining method with the bootstrip values of 1000 by MEGA5 (Tamura et al., 2011). The gene structure analysis was done by the online software^1^. Promoter analysis of *GmNARK* was performed using online analysis software of PLACE^2^ and PlantCARE^3^. The region located 2 kb upstream of *GmNARK* coding sequences was used as promoter sequence for analyzing *cis*-elements. GmNARK protein domain analysis was done by the online software of SMART^4^ and ExPASy^5^. The amino acid alignment was done by the online software of WebLogo 3.^6^

### Gene Expression Analysis

Total RNA was extracted from 7-day-old *Arabidopsis* seedlings and the soybean samples that had been subjected to NaCl or ABA treatment using TRIzol reagent. The RNA samples were used for cDNA synthesis using a cDNA synthesis Supermix with gDNA remover kit (Transgen Biotech, China) following the manufacturer’s instructions. qRT-PCR was carried out using the ABI 7500 real-time PCR system, and SYBR Green qPCR Supermix was used (Invitrogen, Carlsbad, CA, United States). The transcript abundance was calculated by the comparative *C_T_* (cycle threshold) method (*ΔΔC_T_*), and *AtACTIN2* and *GmELF* were used as the internal control for *Arabidopsis* and soybean, respectively. The qRT-PCR experiments were carried out three times, each with three replicates. The primers used in this study were listed in Supplementary Table S1.

### Vector Construction and *Arabidopsis* Transformation

To further analyze the function of *GmNARK* in *Arabidopsis*, we cloned the protein coding region of *GmNARK* by PCR, and inserted the sequence into pTF101 vector, which contained a *Bar* gene as the selecting marker. The enzyme site *SmaI* and *BamHI* were used for the vector construction to generate transgenic plants with *GmNARK* overexpression. *Arabidopsis* transformation was carried out using the floral dip method with *Agrobacterium tumefaciens* strain EHA101 (Clough and Bent, 1998). The *Agrobacteria* containing *35S-GmNARK-pTF101* plasmid was activated with YEP liquid medium shaking overnight and centrifuged to collect the *Agrobacteria*. The *Agrobacteria* was used to infect the inflorescence after diluted to 0.8 (OD_600_) with the transformation solution containing 0.02% Silwet L-77. Transgenic seeds were screened by sowing on MS medium containing 0.001% BASTA. Transgenic homozygous lines were used for the gene expression assay and phenotypic analysis.

### Subcellular Localization Analysis

For the subcellular localization assay, the coding regions of *GmNARK* was inserted into pTF101 vector and the recombinant construct was transformed into the *Agrobacterium tumefaciens* strain EHA101. The *Agrobacteria* EHA101 containing *35S-GmNARK-pTF101* plasmid was activated with YEP liquid medium shaking overnight and centrifuged to collect the *Agrobacteria*. The *Agrobacteria* was used to inject into the *Nicotiana benthamiana* leaves after diluted to 0.2 (OD_600_) with 10 mM MgCl_2_ containing 200 μM AS. The GFP signals were observed using a Leica TCS SP5 confocal laser scanning microscope at 2 days after infiltration. To control for nuclear localization, tissue samples were stained with 100 ng/mL DAPI (4′, 6-diamidino-2-phenylindole).

### FM4-64 Staining

To confirm that GmNARK located at cellular membrane, tobacco leaves expressed fusion protein GmNARK-GFP were immersed into 50 mM FM4-64 (Molecular Probes, Eugene, OR, United States) for 0.5–1 h at darkness. The samples were used to analyze the merged fluorescent signal by Leica TCS SP5 confocal laser scanning microscope.

### Statistical Analysis

All data were analyzed using SigmaPlot 10.0 (Systat Software, Inc., Chicago, IL, United States) and SPSS 16.0 software. The averages and standard deviations were calculated, and for multiple groups of samples, the one-way ANOVA followed by the Dunnett test was used for *P*-value generation between wild type and each transgenic line.

## Results

### Sequence Comparison and Analysis of *GmNARK* and Its Homologues

**FIGURE 1:**
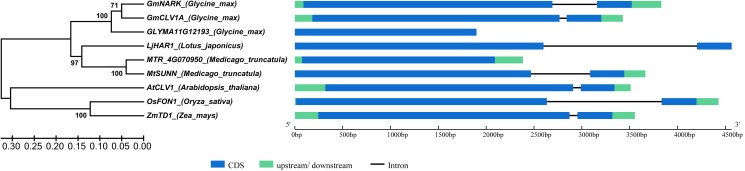
Phylogenetic tree of GmNARK and its homologs in related species. The homologs of *GmNARK* in related species were obtained by the analysis in website of PLAZA 3.0. The gene sequences were downloaded from the JGI database and each of gene locus names were as follows: *GmNARK* (*Glyma.12G040000*), *GmCLV1A* (*Glyma.11G114100*), *GLYMA11G12193* (*Glyma.11G114200*), *LjHAR1* (*chr3.CM0091.1690.r2.m*, the NCBI identity document), *MTR_4g070950* (*Medtr4g070950*), *MtSUNN* (*Medtr4g070970*), *AtCLV1* (*AT1G75820*), *OsFON1* (*LOC_Os06g50340*), *ZmTD1* (*GRMZM2G300133*). Bootstrap consensus of the phylogenetic tree inferred from 1000 replicates using the neighbor-joining method.

A phylogenetic tree was generated using protein sequence of GmNARK and its homologues in related species; eight homologous proteins were retrieved. The genomic sequence length of *GmNARK* and its homologs varied from 1899 to 4566 bp. *GmNARK* contained two exons and one intron harboring the same gene structure with the other selected genes except *GLYMA11G12193* and *MTR_4g070950*, which lacked the second exon in their gene sequences (**Figure 1**). In the phylogenetic tree two clades were obtained. The first clade gathered all the selected leguminous species: *Glycine max*, *Lotus japonicus*, and *Medicago truncatula*. Alignment of deduced protein sequences showed GmNARK shared 86.93 and 52.87% identity with homologues of GmCLV1A (*Glyma.11G114100*) and GLYMA11G12193 (*Glyma.11G114200*) in soybean, respectively (**Figure 1** and Supplementary Figure S1). There were 77.24, 45.46, and 73.56% protein sequence identity shared between GmNARK and LjHAR1 (*chr3.CM0091.1690.r2.m*, the NCBI identity document) in *L. japonicus*, MTR_4g070950 (*Medtr4g070950*) and MtSUNN (*Medtr4g070970*) in *M. truncatula*, respectively (**Figure 1** and Supplementary Figure S1). The second clade of the phylogenetic tree including AtCLV1 (*AT1G75820*) in *Arabidopsis thaliana*, OsFON1 (*LOC_Os06g50340*) in *Oryza sativa* and ZmTD1 (*GRMZM2G300133*) in *Zea mays* (**Figure 1** and Supplementary Figure S1). GmNARK shared 77.24% protein sequence identity with AtCLV1, and 52.97 and 53.09% protein sequence identity with OsFON1 and ZmTD1, respectively (**Figure 1** and Supplementary Figure S1). The result indicated that *NARK* is a conserved gene in monocots and dicots.

### The Promoter of *GmNARK* Contains Manifold *Cis* Regulatory Elements Response to Hormones and Stresses

**FIGURE 2:**
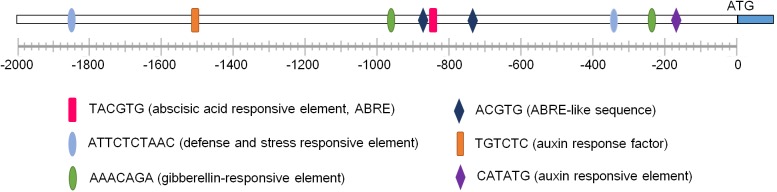
The promoter analysis of *GmNARK*. The 2 kb DNA fragment upstream of the ATG staring code of *GmNARK* were analyzed using online analysis software PLACE (http://www.dna.affrc.go.jp/PLACE/) and PlantCARE (http://bioinformatics.psb.ugent.be/webtools/plantcare/html/).

To understand transcription characteristics and functions of *GmNARK*, we chose the region located 2 kb upstream of *GmNARK* coding sequence as the promoter sequence for *cis*-elements analysis. The promoter analysis of *GmNARK* was performed using online analysis software PLACE^7^ and PlantCARE^8^. The result showed that there were two defense and stress responsive elements (ATTCTCTAAC), one ABRE (TACGTG) *cis*-element, two ABRE-like sequences (ACGTG), two gibberellin responsive elements (AAACAGA) and two auxin responsive elements in its promoter, indicating *GmNARK* may respond to plant hormones (such as ABA, GA and auxin) and abiotic stresses (**Figure 2**). These observations suggest that *GmNARK* plays an important role in plant response to ABA and other abiotic stresses.

### *GmNARK* Was Induced by ABA and NaCl Treatment in Both Shoot and Root

**FIGURE 3:**
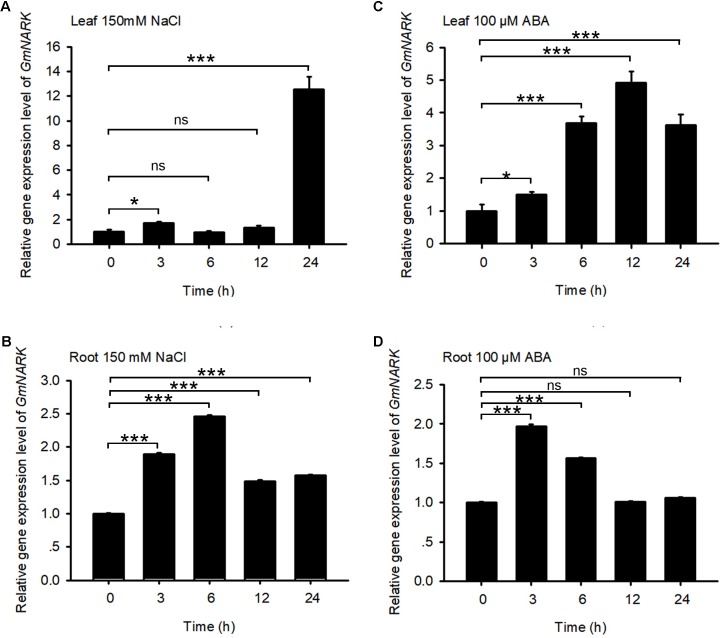
Expression Pattern of *GmNARK.* **(A,B)** *GmNARK* expression assay in response to NaCl in shoot and root by quantitative real-time PCR. Total RNA was extracted from 7-day-old wild-type soybean seedlings treated with 150 mM NaCl at the indicated time points. *GmELF* was used as an internal control. The values are means ± standard error. The Student’s *t*-test was performed and the statically significant treatments were marked with ‘^∗∗∗^’ (*P* < 0.001), ‘^∗^’ (*P* < 0.05) and ‘ns’ (no significance). Three independent biological repeats were performed. **(C,D)** *GmNARK* expression assay in response to ABA in shoot and root by quantitative real-time PCR. Total RNA was extracted from 7-day-old wild-type soybean seedlings treated with 100 μM ABA at the indicated time points. *GmELF* was used as an internal control. The values are means ± standard error. The Student’s *t*-test was performed and the statically significant treatments were marked with ‘^∗∗∗^’ (*P* < 0.001), ‘^∗^’ (*P* < 0.05) and ‘ns’ (no significance). Three independent biological repeats were performed.

The promoter analysis of *GmNARK* revealed that there were ABRE *cis*-elements and stress responsive elements in *GmNARK* promoter, indicating that *GmNARK* may participate in plant response to abiotic stresses in a way that is benefit for plant to adapt to assorted environmental conditions. To verify this hypothesis, we observed the expression level of *GmNARK* in response to different abiotic stresses. 150 mM NaCl and 100 μM ABA treatment were applied to 4-day-old soybean seedlings. The shoot and root tissues were collected at 0, 3, 6, 12, and 24 h, respectively. qPCR was used to check the expression levels of *GmNARK* under ABA and NaCl treatment. As shown in **Figure 3**, the expression of *GmNARK* was induced by ABA and NaCl treatment both in shoot and root (**Figure 3**). Under 150 mM NaCl treatment, the expression level of *GmNARK* increased in shoot, with the highest expression level at 24 h after treatment (**Figure 3A**); In root, *GmNARK* was significantly induced, with the highest expression level at 6 h after treatment (**Figure 3B**). Under 100 μM ABA treatment, the expression level of *GmNARK* significantly increased in shoot, with the highest expression level at 12 h after treatment (**Figure 3C**); In root, *GmNARK* was also notably induced, with the highest expression level at 3 h after ABA treatment (**Figure 3D**). Those results indicate that *GmNARK* is dramatically induced by abiotic stresses, and strongly suggest that it may modulate plant response to abiotic stresses.

### GmNARK Protein Located at Cellular Membrane

**FIGURE 4:**
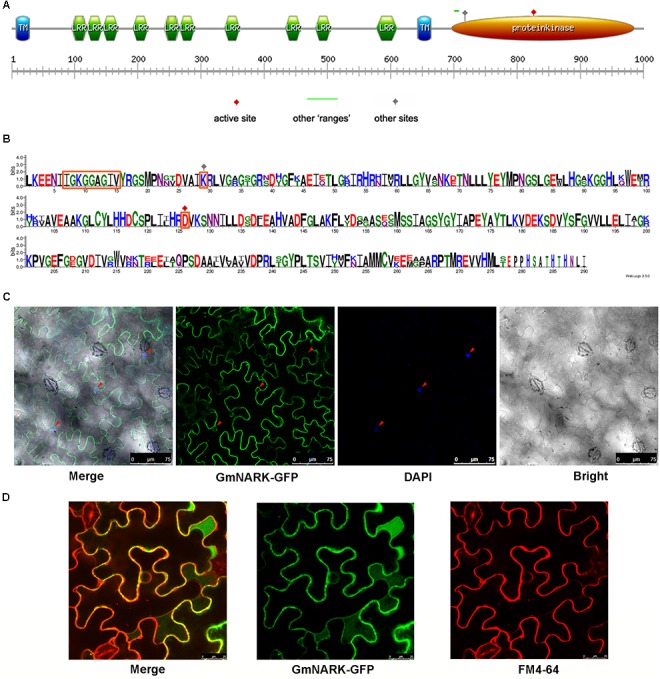
GmNARK protein structure analysis and subcellular location. **(A)** Protein domain analysis of GmNARK. **(B)** Protein sequence alignment between GmNARK and AtCLV1. **(C)** Sub-cellular location analysis of GmNARK-GFP. The image of GmNARK-GFP and DAPI fluorescence in *Nicotiana benthamiana* cells. **(D)** Sub-cellular location analysis of GmNARK-GFP. The image of GmNARK-GFP and membrane staining marker (FM4-64) fluorescence in *N. benthamiana* cells.

The open reading frame of *GmNARK* was 2964 bp, which encoded 987 amino acids with a predicted molecular weight of 108.9 KD and a predicted pI (isoelectric point) of 8.40. To predict the function of GmNARK protein, we analyzed the putative protein domain of GmNARK. Similar to *Arabidopsis* homolog, GmNARK contains two transmembrane domains, ten LRR (Leucine Rich Repeat) domains and one protein kinase domain (**Figure 4A**). The transmembrane domain may be responsible for its cellular location at membrane, while the LRR domains may function in protein-protein reaction. The kinase domain was the key site for GmNARK activity. The Aspartic acid (D) at 821 of the protein sequence was predicted to be the active site for its kinase activity, and the Lysine (K) at 724 may be the other site for its kinase activity (**Figures 4A,B**). Amino acid sequence “IGKGGAGIV” between 702 and 710 may also show significant kinase activity (**Figures 4A,B**). We also made the sequence alignment of kinase domain between GmNARK and AtCLV1, the amino acid length of GmNARK kinase domain was 290, which was 13 amino acid longer than that of AtCLV1. There was high similarity between the two amino acid sequences, especially at the site related to the protein kinase activity (**Figure 4B**).

To confirm the subcellular location of GmNARK we fused GmNARK to green fluorescent protein (GFP) in pTF101-GFP vector and expressed the fusion protein in *Nicotiana benthamiana* transiently. The result showed that the fluorescent signal could be detected at membrane (**Figure 4C**). We also stained the nucleus with 4′, 6-diamidino-2-phenylindole (DAPI); this was used as the nuclear localization marker. Our results showed that the GFP signal could not merge the blue fluorescent signal of DAPI suggesting that GmNARK does not appear at nucleus (**Figure 4C**). To further confirm GmNARK position at membrane we also stained the cellular membrane with FM4-64 (a lipophilic styryl dye used as the membrane localization marker), the GmNARK-GFP signal could merge the FM4-64 red fluorescent signal, providing additional evidence that GmNARK is located at the cell membrane (**Figure 4D**).

### GmNARK Could Form Vesicles for Trafficking via the Endocytic Pathway

**FIGURE 5:**
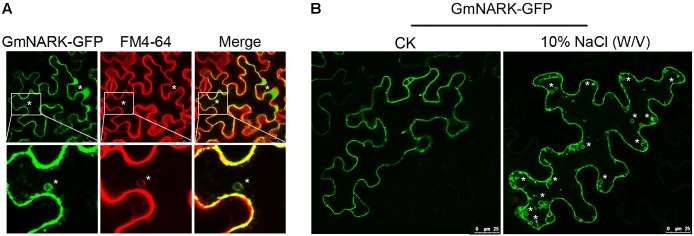
GmNARK-GFP recycles between the plasma membrane and plasma membrane via the endocytic pathway. **(A)** Colocalization of GmNARK-GFP with FM4-64 (endocytic tracer dye) in vesicles. Epifluorescence confocal images were obtained 48 h after agroinfiltration of tobacco epidermal cells with constructs encoding GmNARK-GFP. “^∗^” indicate colocalization of GmNARK-GFP and FM4-64. **(B)** NaCl-induced vesicles formation of GmNARK. Epifluorescence confocal images were obtained 5 min after 10% (w/v) NaCl treatment. “^∗^” indicate the vesicles of GmNARK.

The most well-known marker for endocytic pathway is FM4-64, also known as the membrane selective dye (Vida and Emr, 1995; Ueda et al., 2001; Grebe et al., 2003; Bolte et al., 2004). Interestingly, we found that GmNARK-GFP was not only localized at the cell membrane but also generated vesicles in tobacco cells decorated with the fluorescent signal of GmNARK-GFP, allowing the GFP signal to merge with FM4-64 red fluorescent signal (**Figure 5A**). This result suggests that GmNARK trafficks between cell membrane compartments and plasma membrane forming vesicles via the endocytic pathway. As the expression of *GmNARK* was induced dramatically by NaCl, we treated the tobacco leaves by 10% (w/v) NaCl for 5 min. As shown in **Figure 5B**, the leaf cell was filled with much more vesicles decorated with the fluorescent signal of GmNARK-GFP comparing with the CK indicating that NaCl could induce the vesicles formation of GmNARK (**Figure 5B**). Those results revealed that GmNARK may function by the means of endocytic pathway, and this process was induced greatly by NaCl treatment.

### Overexpression of *GmNARK* Enhanced the Sensitivity of Transgenic *Arabidopsis* to Salt Stress

**FIGURE 6:**
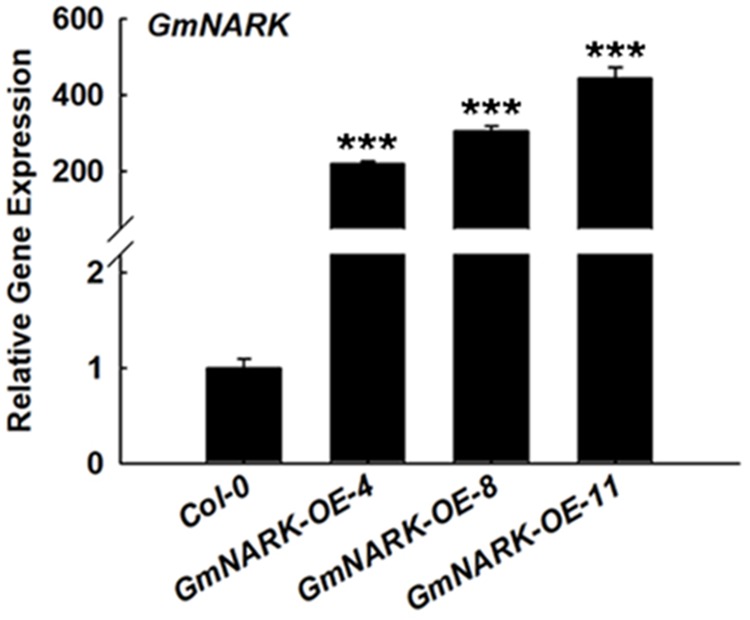
The gene transcript level of *GmNARK* in *GmNARK* overexpression seedlings in *Arabidopsis thaliana*. The gene transcript level of overexpression of *GmNARK*, RNA was extracted from the 7-day-old Arabidopsis transgenic seedlings grown on MS medium, three independent experiments were performed with similar results, each with three replicates. The Student’s *t*-test was performed and the statically significant treatments were marked with ‘^∗∗∗^’ (*P* < 0.001).

To analyze the biological function of GmNARK, we expressed a GmNARK-GFP fusion protein under the control of the CaMV 35S promoter in *Col-0* plants using the *Agrobacterium tumefaciens* mediated floral-dip method. Primary transgenic (T_0_) lines were selected by glyphosate due to the *Basta* gene in the vector of pTF101. Three homozygous lines (*GmNARK-OE-4*, *GmNARK-OE-8*, *GmNARK-OE-11*) were selected for further analysis. The real-time quantitative PCR assay result showed that all the three *GmNARK* overexpression lines exhibited high expression levels of *GmNARK* (**Figure 6**). This result suggested that we successfully overexpressed *GmNARK* in *Arabidopsis* plant, and we will use those three lines for further phenotypic characterizations.

**FIGURE 7:**
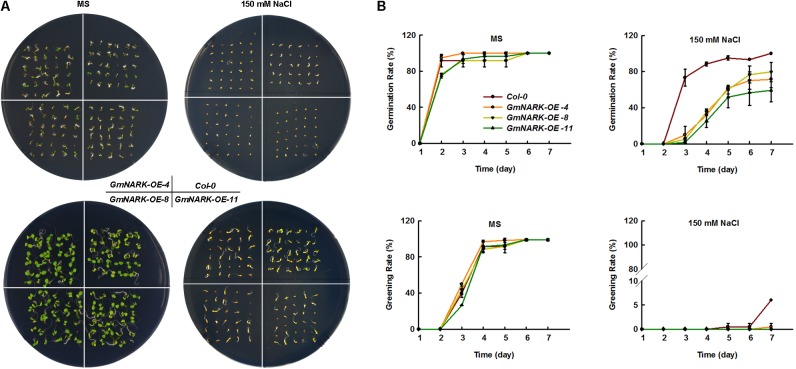
Overexpression of GmNARK increased salt stress sensitivity of transgenic *Arabidopsis* during germination and greening. **(A)** Phenotypic analysis of *Col-0*, *GmNARK-OE-4*, *GmNARK-OE-8*, and *GmNARK-OE-11* treated with 150 mM NaCl. The up panel is the germination images of 3-day-old seeds and the bottom panel is the greening images of 7-day-old seeds. **(B)** The germination rates and greening rates of *Col-0*, *GmNARK-OE-4*, *GmNARK-OE-8*, and *GmNARK-OE-11* under 150 mM NaCl treatment. The data were given as means plus the standard deviation of three independent replicates.

To elucidate whether *GmNARK* was involved in plant response to salt stress, the seeds germination rate and greening rate were measured under NaCl treatment. When the seeds were sown on MS medium containing 150 mM NaCl, the germination rate of transgenic lines overexpressing *GmNARK* were significantly lower than that of the wild type. At the third day after sowing the seeds, the germination rates of three transgenic lines were no more than 20%, while the germination rate of *Col-0* was nearly 75%. However, no difference in the germination rate was found between *Col-0* and transgenic lines under normal growth conditions (**Figures 7A,B**). Similar phenotype was found about the greening rate. On the MS medium of normal conditions, there was nearly no difference about the greening rates between *Col-0* and the three transgenic lines. Under 150 mM NaCl treatment, the greening rates of three transgenic lines *GmNARK-OE-4*, *GmNARK-OE-8*, and *GmNARK-OE-11* were significantly lower than that of *Col-0*. At the seventh day, about 7% *Col-0* seeds were greening, while nearly no greening seeds were found for the three transgenic lines (**Figures 7A,B**). Together, those results suggested that overexpressing *GmNARK* enhanced the transgenic lines sensitivity to salt stress.

### Overexpression of *GmNARK* Enhanced the Sensitivity of Transgenic *Arabidopsis* to ABA

**FIGURE 8:**
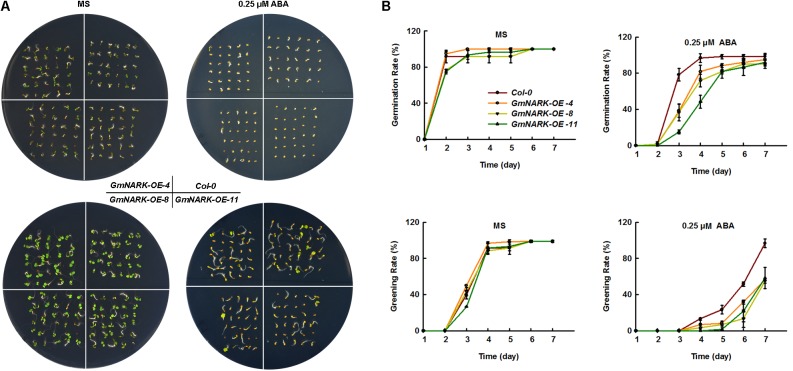
Overexpression of GmNARK increased transgenic *Arabidopsis* sensitivity to ABA during germination and greening. **(A)** Phenotypic analysis of *Col-0*, *GmNARK-OE-4*, *GmNARK-OE-8* and *GmNARK-OE-11* treated with 0.25 μM ABA. The up panel is the germination images of 3-day-old seeds and the bottom panel is the greening images of 5-day-old seeds. **(B)** The germination rates and greening rates of *Col-0*, *GmNARK-OE-4*, *GmNARK-OE-8*, and *GmNARK-OE-11* under 0.25 μM ABA treatment. The data were given as means plus the standard deviation of three independent replicates.

Since *GmNARK* expression level increased under ABA treatment, we next wondered whether *GmNARK* was involved in plant response to ABA. To this end, we germinated the seeds of *Col-0*, *GmNARK-OE-4*, *GmNARK-OE-8*, and *GmNARK-OE-11* on the MS medium containing 0.25 μM ABA or not and tested the plant response to ABA during germination and greening stages. In normal condition, the germination rate and greening rate of *Col-0* were comparable to the transgenic lines (**Figures 8A,B**). By contrast, under ABA treatment, the germination and greening rates of the three transgenic lines were significantly lower than that of the wild type at 3 and 7 days, respectively (**Figures 8A,B**). At the 3 days after sowing the seeds, the germination rates of three transgenic lines were no more than 40%, while the germination rate of *Col-0* was nearly 80%. At the 7 days, about 100% *Col-0* seeds were greening, while nearly 60% greening seeds were found for the three transgenic lines (**Figures 8A,B**). The results suggested *GmNARK* was involved in plant response to ABA during early development.

### Overexpression of *GmNARK* Affected the Expression of ABA Responsive Genes

**FIGURE 9:**
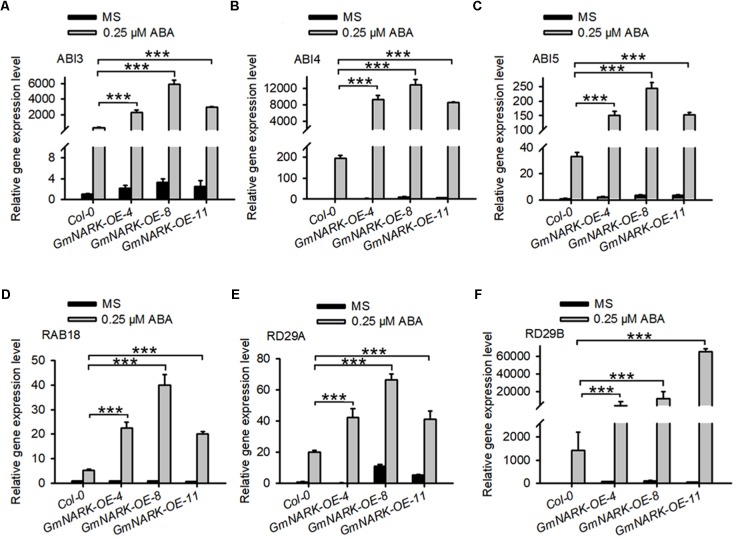
Expression analysis of abiotic stress related genes in the wild type and transgenic plants overexpressing *GmNARK*. The relative transcript level of **(A)** *ABI3*, **(B)** *ABI4*, **(C)** *ABI5*, **(D)** *RAB18*, **(E)** *RD29A*, and **(F)** *RD29B* in *Col-0*, *GmNARK-OE-4*, *GmNARK-OE-8*, and *GmNARK-OE-11* plants were analyzed. Seedlings were grown on MS medium with or without 0.25 μM ABA for 7 days. Three independent experiments were performed with similar results, each with three replicates. *ACTIN2* was used as the internal control. The student’s *t*-test was performed and the statically significant treatments were marked with ‘^∗∗∗^’ (*P* < 0.001).

Given that overexpression of *GmNARK* in *Arabidopsis* positively regulated ABA signaling, we predicted that *GmNARK* may positively regulate the expression of ABA responsive genes. To confirm this, we analyzed the expression of *ABI3*, *ABI4*, *ABI5*, *RAB18*, *RD29A*, and *RD29B*. The seedlings of 7-day-old *Col-0*, *GmNARK-OE-4*, *GmNARK-OE-8*, and *GmNARK-OE-11* under 0.25 μM ABA treatment or not were collected for total RNA extraction and gene expression analysis. In *Col-0*, the expression of all the genes increased under ABA treatment, which was consistent with previous documented results (Lang and Palva, 1992; Yamaguchi-Shinozaki and Shinozaki, 1993; Brocard et al., 2002). In the three transgenic lines all the genes tested were induced under ABA treatment, however, the transcript levels of those genes significantly increased compared with that in *Col-0* under ABA treatment (**Figure 9**). These results prove the positive role of *GmNARK* in ABA signaling.

## Discussion

High salinity is one of the most serious challenges that plants need to cope with and usually results in the repression in plant development leading to limitations in crop yield (Allakhverdiev et al., 2000; Zhu, 2002, 2016). ABA is well-known as an abiotic stress hormone. To cope with serious environmental conditions such as high salinity many adaptive strategies were evolved in plants such as ABA accumulation, which regulates plant response to stress (Zhu, 2002, 2016; Broun, 2004; Bohnert et al., 2006). Soybean is famous for its high protein and oil content, which could be made into several kinds of food. However, salt stress significantly disturbs the development of soybean, which leads to the limitation of plant growth and yield (Essa, 2002; Phang et al., 2008; Ren et al., 2016). To overcome the reduction of seed quality and soybean yield, it is necessary to further explore salt tolerant genes and breed salt resistant cultivars. In this study we focused on the LRR receptor kinase GmNARK and investigated its role in plant response to salt and ABA stresses. According to the *GmNARK* promoter analysis, we found there were multiple *cis*-elements involved in plant response to abiotic stresses, such as ABRE. This result indicates that *GmNARK* is probably induced by stress treatment at transcription level and may be involved in plant response to hormone signaling and abiotic stresses (**Figure 2**). In previous studies, most of the *cis*-elements derived from *Arabidopsis* played similar roles in other plant species (Wang et al., 2014; Cai et al., 2017). To confirm this, we analyzed the *GmNARK* expression level under ABA and NaCl treatment. The result showed that the *GmNARK* transcript level increased in shoot and root, indicating that the *cis*-elements were effective in soybean as well as in *Arabidopsis* in accordance with previous studies (Wang et al., 2014; Cai et al., 2017). The transgenic *Arabidopsis* lines overexpressing *GmNARK* showed increased ABA and NaCl sensitivity in both seed germination and post-germination stages, and GmNARK positively regulated the expression of ABA responsive genes in these lines, indicating that GmNARK regulated plant response to salt through an ABA-dependent way (**Figures 6**–**9**).

Phylogenetic tree analysis showed that GmNARK shared 77.24 and 92% protein sequence identity with AtCLV1 and GmCLV1A, respectively (**Figure 1**). According to previous study, *AtCLV1* mRNA was found in the presumptive shoot meristem during embryo development and could not be detected in root meristem (Clark et al., 1993, 1997). Especially, *AtCLV1* expressed in a region comprising a subset of the central stem cells and the inner portion of the organ forming region of the shoot meristem (Clark et al., 1993, 1997). The expression of *AtCLV1* in some specific stem cells ensured its specified roles in the maintenance of shoot and floral meristems (Clark et al., 1997; Schoof et al., 2000). In soybean, *GmCLV1A* expressed in both root and shoot, including shoot tips (Mirzaei et al., 2017). The mutant of *GmCLV1A* (S562L) performed altered nodal identity, as well as abnormal flower and pod, however, no abnormal nodulation phenotype was found in this mutant (Mirzaei et al., 2017). This result suggests that GmCLV1A plays important roles in regulation of cell division in accordance with the typical functions of AtCLV1 (Mirzaei et al., 2017). In contrast, although *GmNARK* also expressed in both shoot and root, totally different functions were found for this gene in comparison with *AtCLV1* and *GmCLV1A*. In soybean, GmNARK functioned as a LRR receptor kinase in regulating the process of AON (Searle et al., 2003). AON involved a shoot-derived inhibitor (SDI) induced in the shoot by the recognition of GmNARK and the root-derived CLE peptide, and the SDI transported to root to regulate excessive nodulation (Searle et al., 2003; Reid et al., 2011; Lim et al., 2011). In root, GmNARK accepted the CLE peptide induced by nitrate in the root, and inhibited excessive nodule formation (Reid et al., 2011, 2013; Lim et al., 2014; Mirzaei et al., 2017). Although in previous studies GmNARK was involved in JA signaling and regulated the plant defense, no detailed functions of this gene were explored except AON (Kinkema and Gresshoff, 2008). Here in this study we found a totally different function of GmNARK in plant response to salt and ABA stress, the first time to analyze the function of GmNARK involved in abiotic stress response. Taken together, our results indicated that overexpression of GmNARK in *Arabidopsis* increased the plant tolerance to ABA and salt stresses, indicating this gene as an exemplary candidate to breed stress tolerant cultivars..

Recent experimental evidence suggests endocytosis plays an important role in signaling transduction. One way is to integrate membrane localized protein into endosome and transport it to vacuole for degradation. For example, the *Arabidopsis LRR-RLK FLAGELLIN SENSING2* (*FLS2*) can be translocated to endosomes in endocytosis pathway and eventually degraded in vacuole, resulting in the termination of the MAMP-induced defense reaction (Serrano et al., 2007; Geldner and Robatzek, 2008). Another function of endocytosis was its contribution for the recycling of membrane proteins, the dynamic movement and accumulation of receptor proteins in different sites was helpful for rapid response to extracellular signal molecules (Geldner and Robatzek, 2008). The endocytosis extended the limited signaling surface of plant membrane and ensured an efficient cellular signaling system (Geldner and Robatzek, 2008). FM4-64 was frequently used to monitor the endocytic pathway, it contains a lipophilic stain which can bind to lipid component of cells; under excitation the fluorescence can be detected by microscope (Boutte, 2004). In this study, the green fluorescence derived from the fusion protein of GmNARK and GFP was observed in FM4-64 marked vesicles (**Figure 5**). This result suggests that GmNARK is involved in endocytic pathway. As the homolog of GmNARK in *Arabidopsis*, AtCLV1 is also involved in the endocytic pathway. When the ligand protein AtCLV3 bound to AtCLV1 it induced AtCLV1 to transport toward lytic vacuoles and finally be degraded (Nimchuk et al., 2011). We were able to demonstrate that GmNARK was located in the nuclear envelop, suggesting that GmNARK may also be involved in signaling transduction from cell membrane and cytoplasm to nucleus (**Figure 4**). In *Arabidopsis*, calcineurin B-like protein 10 (CBL10) functioned as calcium sensor and it was responsible for plant tolerance to salt stress in the manner of interaction with CIPK24 (SOS2); this protein also existed in fast-moving punctate structures (Kim et al., 2007). Similar to CBL10, the endocytosis location of GmNARK may be related to the fast signal transduction in plant cells under salt stress.

## Author Contributions

CC and ZC conceived the project, designed the experiments, and prepared the manuscript. CC, ZC, CL, DW, and LZ performed the experiments and analyzed the data.

## Conflict of Interest Statement

The authors declare that the research was conducted in the absence of any commercial or financial relationships that could be construed as a potential conflict of interest.

## Footnotes

1 http://gsds.cbi.pku.edu.cn/

2 http://www.dna.affrc.go.jp/PLACE/

3 http://bioinformatics.psb.ugent.be/webtools/plantcare/html/

4 http://smart.embl-heidelberg.de/

5 http://prosite.expasy.org/prosite.html

6 http://weblogo.berkeley.edu/

7 http://www.dna.affrc.go.jp/PLACE/

8 http://bioinformatics.psb.ugent.be/webtools/plantcare/html/
