# Supplementary material for: The Soybean GmNARK Affects ABA and Salt Responses in Transgenic Arabidopsis thaliana
Source: Front Plant Sci. 2018 Apr 18;9:514. doi: 10.3389/fpls.2018.00514 (PMC5915533; doi:10.3389/fpls.2018.00514)
Supplement: Supplementary file 1 [file Table_1.DOCX]

***Supplementary Material***

**The Soybean GmNARK Affects Abiotic Responses in Transgenic *Arabidopsis thaliana***

Chunhong Cheng, Changman Li, Diandong Wang, Lifeng Zhai, Zhaoming Cai^*^

College of Life Science and Technology

Yangtze Normal University

Chongqing 408100, P.R. China

^*^Corresponding author: [caizhaoming-2000@163.com](mailto:xli@mail.hzau.ac.cn)

**Supplementary Information**

**Supplementary Figure S1** The protein sequence alignment of GmNARK and its homologues in related species.

**Supplementary Table S1** The primers used in this study.


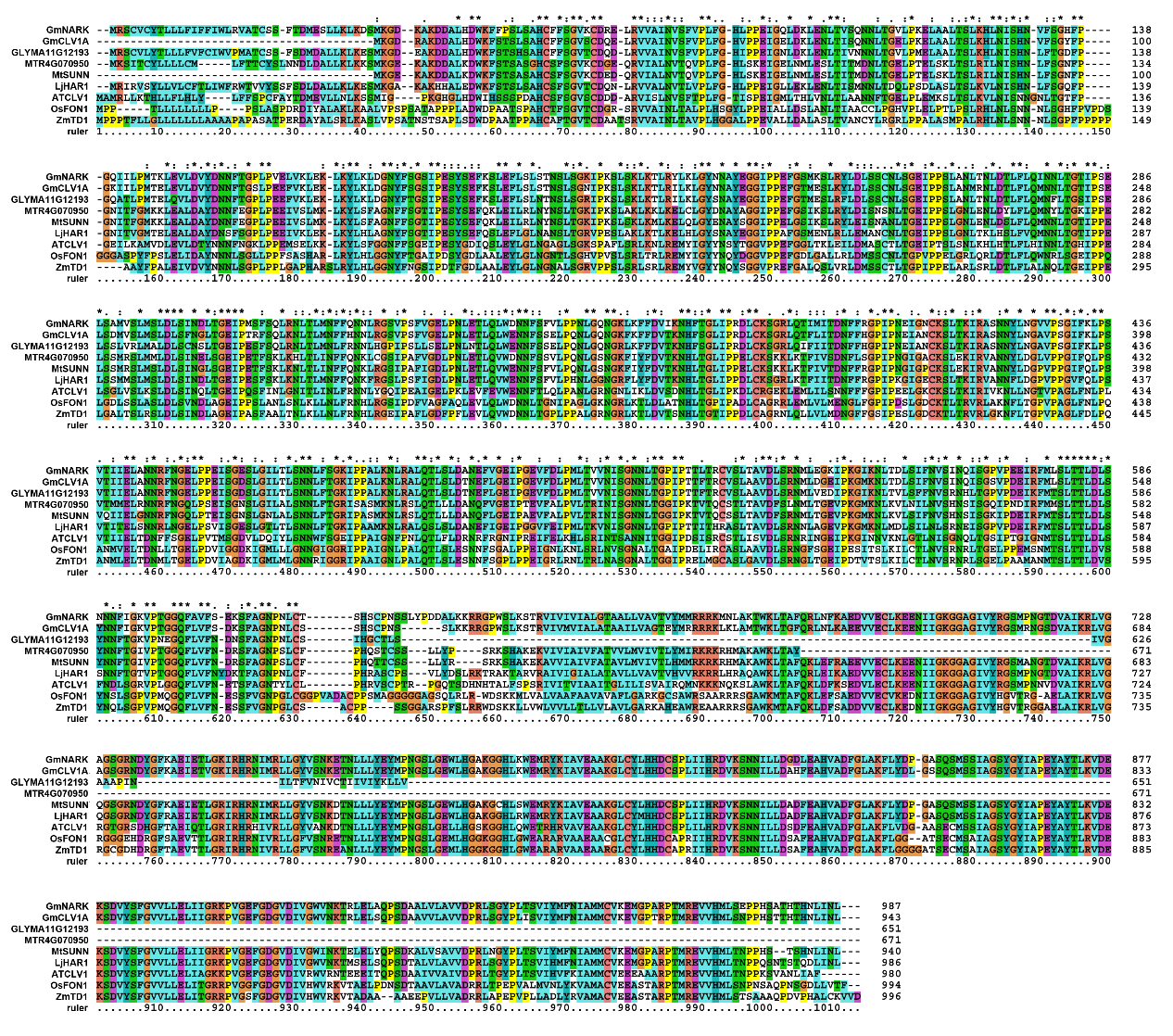


**Supplementary Figure S1** The protein sequence alignment of GmNARK and its homologues in related species. The clustalx software was used for this alignment.

| **Supplemental table 1** **The primers used in this study** | | |
| --- | --- | --- |
| **Name** | **DNA sequence（5’-3’）** | **Purpose** |
| **GmNARK-OE-F** | CCCCCGGGATGAGAAGCTGTGTGTGCT | vector construction |
| **GmNARK-OE-R** | CGGGATCCCTAGAGATTAATTAGGTTGTGAG | vector construction |
| **qRT- GmNARK-F** | GAGTTTGGAGACGGGGTGG | qRT PCR |
| **qRT- GmNARK-R** | CCACTGCCAACACCAACG | qRT PCR |
| **GmELF1B-F** | GTTGAAAAGCCAGGGGACA | qRT PCR |
| **GmELF1B-R** | TCTTACCCCTTGAGCGTGG | qRT PCR |
| **ACTIN2 qRT F** | GCCATCCAAGCTGTTCTCTC | qRT PCR |
| **ACTIN2 qRT R** | GCTCGTAGTCAACAGCAACAA | qRT PCR |
| **ABI3 qRT F** | CACAGCCAGAGTTCCTTCCTTTACT | qRT PCR |
| **ABI3 qRT R** | TAGTTGCTGAGGAACACAAACGG | qRT PCR |
| **ABI4 qRT F** | GGGCAGGAACAAGGAGGAAGTG | qRT PCR |
| **ABI4 qRT R** | TCTCCTCCAAAAGGCCAAATGGT | qRT PCR |
| **ABI5 qRT F** | ATGATCAAGAACCGCGAGTCTGC | qRT PCR |
| **ABI5 qRT R** | CGGTTGTGCCCTTGACTTCAAAC | qRT PCR |
| **RAB18 qRT F** | GGC TTG GGA GGA ATG CTT CA | qRT PCR |
| **RAB18 qRT R** | CGC TTG AGC TTG ACC AGA CT | qRT PCR |
| **RD29A qRT F** | GGAAGTGAAAGGAGGAGGAGGAA | qRT PCR |
| **RD29A qRT R** | CACCACCAAACCAGCCAGATG | qRT PCR |
| **RD29B qRT F** | GAATCAAAAGCTGGGATGGA | qRT PCR |
| **RD29B qRT R** | TGCTCTGTGTAGGTGCTTGG | qRT PCR |
